# Clinical characteristics, risk factors and antiviral treatments of influenza in immunosuppressed inpatients in Beijing during the 2015–2020 influenza seasons

**DOI:** 10.1186/s12985-021-01739-1

**Published:** 2022-01-15

**Authors:** Yafen Liu, Yue Wang, Huan Mai, YuanYuan Chen, Baiyi Liu, YiSi Liu, Ying Ji, Xu Cong, Yan Gao

**Affiliations:** 1 Department of Infectious Diseases, Peking University Hepatology Institute, Peking University People’s Hospital, No. 11, Xizhimen South Street, Xicheng District, Beijing, 100044 People’s Republic of China; 2 Peking University Hepatology Institute, Peking University People’s Hospital, No. 11, Xizhimen South Street, Xicheng District, Beijing, 100044 People’s Republic of China

**Keywords:** Influenza, Immunosuppression, Malignancy, Haemopoietic stem cell transplantation, Antiviral therapy, Influenza vaccination

## Abstract

**Background:**

Compared with immunocompetent patients, immunosuppressed patients have higher morbidity and mortality, a longer duration of viral shedding, more frequent complications, and more antiviral resistance during influenza infections. However, few data on this population in China have been reported. We analysed the clinical characteristics, effects of antiviral therapy, and risk factors for admission to the intensive care unit (ICU) and death in this population after influenza infections and explored the influenza vaccination situation for this population.

**Methods:**

We analysed 111 immunosuppressed inpatients who were infected with influenza virus during the 2015–2020 influenza seasons. Medical data were collected through the electronic medical record system and analysed. Univariate analysis and multivariate logistics analysis were used to identify risk factors.

**Results:**

The most common cause of immunosuppression was malignancies being treated with chemotherapy (64.0%, 71/111), followed by haematopoietic stem cell transplantation (HSCT) (23.4%, 26/111). The most common presenting symptoms were fever and cough. Dyspnoea, gastrointestinal symptoms and altered mental status were more common in HSCT patients than in patients with immunosuppression due to other causes. Approximately 14.4% (16/111) of patients were admitted to the ICU, and 9.9% (11/111) of patients died. Combined and double doses of neuraminidase inhibitors did not significantly reduce the risk of admission to the ICU or death. Risk factors for admission to the ICU were dyspnoea, coinfection with other pathogens and no antiviral treatment within 48 h. The presence of dyspnoea and altered mental status were independently associated with death. Only 2.7% (3/111) of patients less than 12 months old had received a seasonal influenza vaccine.

**Conclusion:**

Fever and other classic symptoms of influenza may be absent in immunosuppressed recipients, especially in HSCT patients. Conducting influenza virus detection at the first presentation seems to be a good choice for early diagnosis. Clinicians should pay extra attention to immunosuppressed patients with dyspnoea, altered mental status, coinfection with other pathogens and no antiviral treatment within 48 h because these patients have a high risk of severe illness. Inactivated influenza vaccines are recommended for immunosuppressed patients.

## Background

Influenza infection receives continuous attention due to the associated significant morbidity and mortality worldwide. Approximately 29,0000–65,0000 seasonal influenza-associated respiratory deaths are estimated each year globally [1]. In China, approximately 88,100 influenza-associated respiratory deaths occur annually [2]. The number of immunosuppressed patients, such as patients with malignancies receiving chemotherapy, patients who have undergone haemopoietic stem cell transplantation (HSCT) or solid-organ transplantation (SOT), patients on chronic haemodialysis, and patients receiving systemic corticosteroids, has increased each year [3, 4]. Compared with immunocompetent patients, immunosuppressed patients have higher morbidity and mortality, a longer duration of viral shedding, more frequent complications, and more antiviral resistance, increasing the potential for nosocomial transmission [3, 5–9].

Previous studies mostly focused on influenza infection in patients who have undergone HSCT and SOT, paying less attention to patients with immunosuppression due to other causes [9–11]. In addition, few studies on influenza virus infections in immunosuppressed patients in China have been reported. Neuraminidase inhibitors such as oseltamivir and peramivir are the mainstays of antiviral therapy. A previous study suggested high doses and a long duration of antiviral treatment for patients who were immunocompromised [7, 12]; however, specific data on the effect of antiviral therapy in immunosuppressed patients in China are rarely reported. Furthermore, influenza vaccine responses in immunosuppressed patients were evaluated [3]. Thus, we analysed the clinical characteristics of and risk factors and antiviral treatments for influenza in immunosuppressed inpatients in Beijing during the 2015–2020 influenza seasons and explored influenza vaccination strategies in immunosuppressed patients.

## Materials and methods

### Patients and study design

During the 2015–2020 influenza seasons (November to the following March), a total of 45,518 nasal swab specimens were obtained from outpatient and inpatient influenza-like illness patients at Peking University People’s Hospital (PKUPH). PKUPH is a national influenza surveillance sentinel unit, receiving at least 100,000 inpatients from all Beijing districts annually. A total of 8081 samples screened positive for influenza A or/and B viruses by using the colloidal gold method. Among the 251 inpatients, 111 immunosuppressed patients were enrolled in this study. Original samples from the inpatients were collected and immediately placed in virus transport media tubes for analysis. Immunocompromised patients were defined as patients with HIV infection, recipients of solid-organ transplants, recipients of haemopoietic stem cell transplants, patients with malignancies receiving chemotherapy, patients on chronic haemodialysis, and patients receiving systemic corticosteroids [3]. Influenza infections were defined as samples that tested positive for influenza virus by using the reverse-transcription polymerase chain reaction (RT–PCR) method [13].

### Data collection

Data on demographic factors (age, sex, the cause of immunosuppression, comorbidity, smoking, rejection in the preceding 3 months, antilymphocyte globulin in the preceding 6 months, interleukin-2 receptor antagonists in the preceding 6 months, monoclonal antibody in the preceding 6 months, the use of corticosteroids in the preceding 3 months, seasonal influenza vaccination in the preceding 12 months, the use of neuraminidase inhibitors before admission), clinical presentation and complications (fever, sore throat, rhinorrhoea, cough, headache, muscle soreness, dyspnoea, gastrointestinal symptoms, altered mental status, symptom onset, coinfection with other pathogens, complications, antiviral treatment, admission to the intensive care unit, mechanical ventilation, virus detection turned negative days, death) and laboratory test results [white blood cell (WBC), neutrophil, lymphocyte, and platelet counts; alanine aminotransferase; aspartate aminotransferase; albumin; lactate dehydrogenase; creatine kinase; blood creatinine; FiO_2_; lactic acid; erythrocyte sedimentation rate; C-reactive protein] were collected through medical records. The comorbidities included diabetes, coronary heart disease, cardiac insufficiency, cerebrovascular disease, chronic nephropathy, chronic hepatopathy, and chronic lung disease based on specialist diagnosis. Laboratory test results were obtained from a nationally accredited laboratory.

### RNA extraction and verifying viral infection using RT–PCR

RNA was extracted from samples using the QIAamp Viral RNA Mini Kit (Cat. No. 52904, Qiagen, Hilden, Germany), and the extracted RNA was used as a template to perform RT–PCR with a commercial kit (Cat. No.18080051, Invitrogen, Carlsbad, CA, USA). Total RNA (8 μl), universal primer 5’-AGCAAAAGCAGG-3’ (4 μl) and 10 mM dNTP (1 μl) were added to an reverse-transcription tube on ice, incubated at 65 °C for 5 min and then chilled on ice again for at least 1 min. Thereafter, 10 × RT buffer (2 μl), 25 mM MgCl_2_ (4 μl), 0.1 M DTT (2 μl), RNase inhibitor (1 μl of 40 U/µL) and SuperScript® III reverse transcriptase (1 μl of 200 U/µL) were added to the tube, which was then incubated at 50 °C for 50 min, followed by 85 °C for 5 min. RNase H (1 μl) was added to each tube, and the tubes were then incubated for 20 min at 37 °C after chilling on ice and brief centrifugation. We verified the presence of the virus using PCR with high-fidelity thermostable DNA polymerase (Cat. No. 11304011, Invitrogen) with specific primers as follows: forward primers (5′-ACATTCGAAGCAACTGGAAA-3′ and 5′-ACCCTCAGTGTGATGGCTTCCAAA-3′), reverse primers (5′- GTRTTRCAATCGTGGACTGG-3′ and 5′- TAAGGGAGGCATAATCCGGCACAT-3′) for influenza A(H1N1)pdm09 and H3N2 and forward primer (5′-AGACCAGAGGGAAACTATGCCC-3′) and reverse primer (5′-TCCGGATGTAACAGGTCTGACTT-3′) for influenza B viruses. The PCR amplification system included the cDNA template (4 μl); autoclaved, distilled water (12.1 μl); 10X High Fidelity PCR Buffer (2 μl); 50 mM MgSO_4_ (0.6 μl); 10 mM dNTP Mix (0.4 μl); 10 µM forward primer (0.4 μl); 10 µM reverse primer (0.4 μl); and Platinum® Taq DNA Polymerase High Fidelity (0.1 μl of 5 U/µL). The PCR conditions were 94 °C for 3 min, followed by 35 cycles of 94 °C for 0.5 min, 55 °C for 0.5 min and 72 °C for 1.5 min, with extension at 72 °C for 7 min. The PCR products were identified through electrophoresis.

### Detection of bacteria, fungi and other viruses

Bacteria and fungi were identified from blood, mid-stream clean-catch urine, qualified sputum, bronchoalveolar lavage fluid or ascites using microbiological culture. Qualified sputum originating from the lower respiratory tract was defined as that containing > 25 granulocytes and < 10 epithelial cells per field of view under a low-power microscope. Fungal pathogens were also tested by G (1,3-beta-D-glucan) or GM (galactomannan) tests. Other viruses in blood samples were detected using RT–PCR. All detections were performed at the nationally accredited laboratory of PKUPH.

### Statistical analysis

All statistical analyses were performed using SPSS statistical software version 22.0 (SPSS Inc., Chicago, IL, USA). Categorical variables were expressed as counts (percentage) and compared using the χ^2^ test, while continuous variables were expressed as the means ± SD or median (interquartile range) and compared using an independent-samples t test. For multigroup comparisons, one-way analysis of variance (ANOVA) with a least-squares difference post hoc test was applied. For data not normally distributed, the Mann–Whitney U test was used if only two groups were being compared, and Kruskal–Wallis one-way ANOVA was used if more than two groups were being compared. Univariate analysis and multivariate logistics analysis were used to identify risk factors for admission to the ICU and death. The removal probability for multivariate stepwise logistic regression analysis was 0.10. Results with *P* values < 0.05 were considered statistically significant.

## Results

### Distribution of detected viruses

One hundred and eleven samples from immunosuppressed inpatients who tested positive for influenza virus using the colloidal gold method were verified and classified using RT–PCR assay. Influenza A viruses were detected in 80 samples, and the remaining 31 samples were positive for influenza B viruses.

### Clinical characteristics of the study patients

As shown in Table 1, the most common cause of immunosuppression was malignancies being treated with chemotherapy (64.0%; 71/111), followed by haematopoietic stem cell transplantation (23.4%; 26/111), autoimmune disorders with immunosuppressive therapy (9.0%; 10/111), chronic haemodialysis (2.7%; 3/111), and solid-organ transplantation (0.9%; 1/111). Malignancies included almost all types of tumours, such as lung cancer, bone tumours, hepatomas, cholangiocarcinomas, gastrointestinal tumours, urological tumours, gynaecological tumours, leukaemia, lymphomas, other haematological tumours, breast cancer, thyroid cancer, neuroendocrine neoplasms and retinoblastomas. Among the patients with malignancies, one patient had both breast cancer and leukaemia, and two had both gynaecological tumours and leukaemia. The most common transplantation type was haematopoietic stem cell transplantation. One case of solid-organ transplantation was kidney transplantation. The median time of symptom onset after HSCT was 9 months (range 1–73 months). A total of 69.2% (18/26) of HSCT patients experienced rejection in the preceding 3 months. The kidney transplant patient presented symptoms 15 months after transplantation and had no rejection within the preceding 3 months. The use of corticosteroids in the preceding 3 months was reported in 27.0% (30/111) of patients. Seasonal influenza vaccination in patients within the preceding 12 months old was reported in 2.7% (3/111) of patients. These three patients had no complications or coinfections, and fever disappeared within 48 h after antiviral treatment.

**Table 1:** Demographics of immunosuppressed patients with influenza infections

	Findings (n = 111)
Age, median years (range)	51 (1–92)
Male sex (%)	55 (49.5)
Cause of immunosuppression	
Malignancies and chemotherapy (%)	71 (64.0)
Haematopoietic stem cell transplantation (%)	26 (23.4)
Autoimmune disorders and immunosuppressive therapy (%)	10 (9.0)
Chronic haemodialysis	3 (2.7)
Solid-organ transplantation (%)	1 (0.9)
Comorbidity	
Diabetes (%)	13 (11.7)
Coronary heart disease (%)	4 (3.6)
Cardiac insufficiency (%)	1 (0.9)
Cerebrovascular disease (%)	6 (5.4)
Chronic nephropathy (%)	12 (10.8)
Chronic hepatopathy (%)	3 (2.7)
Chronic lung disease (%)	5 (4.5)
Smoking (%)	21 (18.9)
Rejection after HSCT in the preceding 3 months (%)	18/26 (69.2)
Antilymphocyte globulin in the preceding 6 months (%)	2 (7.7)
Interleukin-2 receptor antagonists in the preceding 6 months (%)	9 (8.1)
Monoclonal antibody in the preceding 6 months (%)	3 (2.7)
Use of corticosteroids in the preceding 3 months (%)	30 (27.0)
Seasonal influenza vaccination in the preceding 12 months (%)	3 (2.7)
Neuraminidase inhibitor use before admission (%)	10 (9.0)

HSCT: haematopoietic stem cell transplantation

As shown in Table 2, the most common presenting symptom was fever in 92.8% (103/111) of patients, followed by cough 50.6% (44/87), muscle soreness 32.4% (22/68), rhinorrhoea 27.4% (20/73), headache 22.5% (16/71), sore throat 22.2% (16/72), dyspnoea 17.1% (13/76), gastrointestinal symptoms 15.4% (12/78), and altered mental status 6.3% (6/95). The medium symptom onset was 24 h (range 2–456 h). A total of 40.5% (45/111) of patients had complications, including pneumonia and other complications. Imaging (chest radiograph or CT) data were available for 98 of 111 patients, and 42.9% (42/98) of patients had imaging findings consistent with pneumonia. Regarding other complications, acute respiratory distress syndrome was reported in 9.0% (10/111) of patients, shock in 6.3% (7/111), acute kidney injury in 3.6% (4/111), and viral encephalitis in 0.9% (1/111). Furthermore, we analysed the top three immunosuppression types. The results showed that dyspnoea, gastrointestinal symptoms and altered mental status were more common in HSCT patients than in patients with immunosuppression due to other causes, with more complications, higher mortality and more hospitalization days. The symptom onset in the HSCT group was 60 h (range 24–456 h) later than that in patients with immunosuppression due to other causes (Table 2).

**Table 2:** Clinical presentation and complications of immunosuppressed patients with influenza infections

	All types (n = 111)	Malignancies (n = 71)	HSCT (n = 26)	Autoimmune disorders (n = 10)
Fever (%)	103/111 (92.8)	69/71 (97.2)	22/26 (84.6)	8/10 (80.0)
Sore throat (%)	16/72 (22.2)	11/50 (22.0)	2/14 (14.3)	2/7 (28.6)
Rhinorrhoea (%)	20/73 (27.4)	8/49 (16.3)	6/16 (37.5)	4/7 (57.1)
Cough (%)	44/87 (50.6)	22/54 (40.7)	16/22 (72.7)	5/9 (55.6)
Headache (%)	16/71 (22.5)	8/48 (16.7)	2/13 (15.4)	5/9 (55.6)
Muscle soreness (%)	22/68 (32.4)	14/49 (28.6)	3/13 (23.1)	3/5 (60.0)
Dyspnoea (%)	13/76 (17.1)	4/51 (7.8)	8/19 (42.1)	0/5 (0.0)
Gastrointestinal symptoms (%)	12/78 (15.4)	6/52 (11.5)	6/19 (31.6)	0/5 (0.0)
Altered mental status (%)	6/95 (6.3)	2/58 (3.4)	3/24 (12.5)	0/10 (0.0)
Symptom onset, hours (range)	24.0 (2.0–456.0)	24.0 (6.0–312.0)	60.0 (24.0–456.0)	24.0 (2.0–96.0)
Coinfection with other pathogens (%)	21/111 (18.9)	10/71 (14.1)	11/26 (42.3)	0/10 (0.0)
Complications (%)	45/111 (40.5)	22/71 (31.0)	17/26 (65.4)	4/10 (40.0)
Pneumonia on chest radiograph or CT scan (%)	42/98 (42.9)	21/61 (34.4)	16/24 (66.7)	4/9 (44.4)
Acute respiratory distress syndrome (%)	10/111 (9.0)	3/71 (4.2)	7/26 (26.9)	0/10 (0.0)
Shock (%)	7/111 (6.3)	2/71 (2.8)	3/26 (11.5)	0/10 (0.0)
Acute kidney injury (%)	4/111 (3.6)	0/71 (0.0)	2/26 (7.7)	1/10 (10.0)
Viral encephalitis (%)	1/111 (0.9)	0/71 (0.0)	1/26 (3.8)	0/10 (0.0)
Admission to the ICU (%)	16/111 (14.4)	8/71 (11.3)	7/26 (26.9)	0/10 (0.0)
Mechanical ventilation (%)	9/111 (8.1)	2/71 (2.8)	7/26 (26.9)	0/10 (0.0)
Virus detection turning negative, days (range)*	3.0 (1.0–11.0)	3.0 (1.0–11.0)	3.0 (1.0–9.0)	7.0 (2.0–9.0)
Number of hospitalization days (range)	15.0 (1.0–130.0)	12.0 (1.0–65.0)	44.0 (1.0–130.0)	10.5 (4.0–56.0)
Death (%)	11/111 (9.9)	4/71 (5.6)	7/26 (26.9)	0/10 (0.0)

*Data are available only for 41 patients; HSCT, haematopoietic stem cell transplantation; ICU, intensive care unit

As shown in Table 3, routine blood test results were characterized by lymphopenia in immunosuppressed patients with influenza infections. The erythrocyte sedimentation rate (median: 31.5) and C-reactive protein (median: 27.7) were slightly higher than normal. In the subgroup analysis, C-reactive protein was higher in patients with malignancies who were receiving chemotherapy than in patients with immunosuppression due to other causes.

**Table 3:** Laboratory test results of immunosuppressed patients with influenza infections

	All types (n = 111)	Malignancies (n = 71)	HSCT (n = 26)	Autoimmune disorders (n = 10)
WBC* (× 10^9^/L)	5.0 (0.0–64.7)	5.2 (0.2–64.7)	3.3 (0.0–13.5)	6.0 (0.9–8.5)
Neutrophils (%)	71.2 (0.0–96.4)	71.2 (0.0–96.4)	71.9 (0.5–96.4)	72.8 (58.0–89.8)
Neutrophils (× 10^9^/L)	3.6 (0.0–63.7)	3.8 (0.0–63.7)	3.0 (0.0–12.7)	4.2 (0.5–7.6)
Lymphocytes (%)	15.6 (1.4–97.4)	15.8 (1.8–97.4)	15.0 (1.4–60.9)	16.6 (3.1–33.8)
Lymphocytes (× 10^9^/L)	0.7 (0.0–58.1)	0.7 (0.0–58.1)	0.7 (0.0–2.0)	0.7 (0.3–1.7)
Platelets (× 10^9^/L)	118.0 (8.0–429.0)	149.0 (8.0–429.0)	78.0 (8.0–217.0)	156.5 (28.0–313.0)
Alanine aminotransferase (U/L)	21.0 (2.0–279.0)	19.0 (6.0–279.0)	22.0 (2.0–182.0)	16.5 (8.0–28.0)
Aspartate aminotransferase (U/L)	22.0 (6.0–198.0)	20.5 (6.0–198.0)	24.0 (11.0–182.0)	24.0 (15.0–45.0)
Albumin (g/L)	33.7 (20.1–48.7)	35.1 (20.1–48.7)	33.1 (24.4–42.5)	35.1 (29.3–36.9)
Lactate dehydrogenase (U/L)	190.0 (77.0–5536.0)	186.0 (77.0–5536.0)	280.0 (169.0–890.0)	168.0 (154.0–229.0)
Creatine kinase (U/L)	29.0 (4.0–725.0)	35.0 (4.0–725.0)	23.0 (6.0–157.0)	19.0 (13.0–29.0)
Blood creatinine (µmol/L)	59.0 (22.0–1189.0)	58.0 (22.0–178.0)	58.5 (23.0–139.0)	59.0 (30.0–84.0)
FiO_2_ (mmHg)	360.9 (97.5–512.6)	323.3 (179.3–457.4)	338.3 (97.5–472.1)	431.7 (350.8–512.6)
Lactic acid (mmol/L)	1.2 (0.4–2.6)	1.1 (0.9–1.6)	1.7 (0.4–2.6)	0.8 (0.6–1.0)
Erythrocyte sedimentation rate (mm/h)	31.5 (9.0–123.0)	32.0 (9.0–123.0)	16.0 (15.0–18.0)	27.5 (16.0–68.0)
C-reactive protein (mg/L)	27.7 (0.5–416.9)	48.3 (0.5–416.9)	12.8 (0.5–139.1)	11.26 (0.5–105.83)

^*^WBC: white blood cell

Coinfections were found in 18.9% (21/111) of patients (Table 4). Bacterial pathogens were identified in blood, urine, qualified sputum, or ascites in 12 (10.8%) of 111 patients, including *Escherichia coli*, *Klebsiella pneumoniae*, *Pseudomonas aeruginosa*, *Stenotrophomonas maltophilia*, *methicillin-resistant Staphylococcus aureus* (MRSA), *Enterococcus faecium* and *Streptococcus*. Two patients had fungal infections (both were *Aspergillus spp.*). The number of hospitalization days were 90 and 51 days, respectively (the latter patient died). Cytomegalovirus and Epstein–Barr virus viremia were detected in 8 (7.2%) and 4 (3.6%) of 111 patients, respectively, while herpes simplex virus was detected in 1 patient, parainfluenza virus in 1 patient and polyomaviruses (JC virus and BK polyomavirus) in 2 patients.

**Table 4:** Distribution of pathogens in patients with coinfections

Patient No	Bacteria (Sample type)	Fungi (Sample type)	Viruses (Blood nucleic acid)
7	*Enterococcus faecium* (Blood)		
12		*Aspergillus spp.*(BAL + GM + chest CT)	Cytomegalovirus
14	*Stenotrophomonas maltophilia* (Qualified sputum)		
20	*Escherichia coli* and *Enterococcus faecium* (Ascites)		
23		*Aspergillus spp.*(BAL + GM + chest CT)	Parainfluenza virus and Cytomegalovirus
26	*Streptococcus* (Qualified sputum)		
35	*Escherichia coli*, *Klebsiella pneumoniae* and *Enterococcus faecium* (Ascites)		
43, 68	*Escherichia coli* (Blood)		
61, 111			Cytomegalovirus
62, 75			Cytomegalovirus and Epstein–Barr virus
63	*Klebsiella pneumoniae* and *Pseudomonas aeruginosa* (Blood)		Epstein–Barr virus
65			Herpes simplex virus
74	*Klebsiella pneumoniae* (Blood)		Cytomegalovirus
77			Epstein–Barr virus
85			JC virus
99	*Stenotrophomonas maltophilia and Enterococcus faecium (Ascites)*		
103	*Escherichia coli* (Blood and urine)		
109	MRSA (Blood)		Cytomegalovirus and BK polyomavirus

BAL: bronchoalveolar lavage fluid; GM: galactomannan; MRSA: methicillin-resistant *Staphylococcus aureus*

**Fig. 1:**
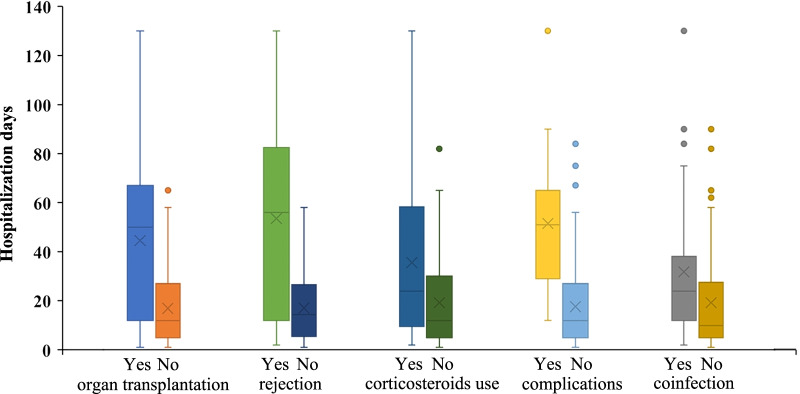
Box plot of the number of hospitalization days in immunosuppressed inpatients with influenza infections. The number of hospitalization days was significantly associated with organ transplantation, rejection in the preceding 3 months, the use of corticosteroids in the preceding 3 months, complications, and coinfection with other pathogens

The median number of hospitalization days was 15 days (range 1–130 days). The number of hospitalization days was significantly associated with organ transplantation, rejection in the preceding 3 months, the use of corticosteroids in the preceding 3 months, complications, and coinfection with other pathogens (Fig. 1). Compared with different immunosuppressive subgroups, the median number of hospitalization days in the HSCT group was 44 days, which was significantly longer than that of patients receiving chemotherapy for malignant tumours and immunosuppression for autoimmune diseases.

### Effects of antiviral therapy

Among the 111 patients, 104 (93.7%) received antiviral treatment (Table 5). In detail, 76.0% (79/104) were given oseltamivir, 8.6% (9/104) were given intravenous peramivir, and the remaining 15.4% (16/104) were given oseltamivir and peramivir. The standard dose of antiviral treatment was given for 89.4% (93/104) of patients. The adult patients received oseltamivir at the equivalent of 75 mg twice or peramivir 300 mg once per day, and children received the standard dose according to weight. Approximately 10.6% (11/104) of patients received a double dose. In the subgroup analysis, more HSCT patients received combined and double doses of neuraminidase inhibitors. However, neither a combined nor a double dose of neuraminidase inhibitors was significantly associated with admission to the ICU or death (*P* > 0.05).

**Table 5:** Antiviral treatment of immunosuppressed patients with influenza infections

	All types (n = 111)	Malignancies (n = 71)	HSCT (n = 26)	Autoimmune disorders (n = 10)
Antiviral treatment (%)	104/111 (93.7)	65/71 (91.5)	25/26 (96.2)	10/10 (100.0)
No antiviral treatment (%)	7/111 (6.3)	6/71 (8.5)	1/26 (3.8)	0/10 (0.0)
Antiviral treatment within 48 h (%)	93/111 (83.8)	60/71 (84.5)	20/26 (76.9)	9/10 (90.0)
Antiviral treatment after 48 h (%)	11/111 (9.9)	5/71 (7.0)	5/26 (19.2)	1/10 (10.0)
Oseltamivir (%)	79/104 (76.0)	52/65 (80.0)	15/25 (60.0)	9/10 (90.0)
Peramivir (%)	9/104 (8.6)	7/65 (10.8)	2/25 (8.0)	0/10 (0.0)
Combination of oseltamivir and peramivir (%)	16/104 (15.4)	6/65 (9.2)	8/25 (32.0)	1/10 (10.0)
Standard dose of neuraminidase inhibitors (%)	93/104 (89.4)	59/65 (90.8)	21/25 (84.0)	9/10 (90.0)
Double dose of neuraminidase inhibitors (%)	11/104 (10.6)	6/65 (9.2)	4/25 (16.0)	1/10 (10.0)

The median duration of antiviral treatment was 6 days (range 1–32). A total of 83.8% (93/111) of patients began antiviral treatment within 48 h after illness onset, and the other 9.9% (11/111) received antiviral treatment after 48 h. No antiviral treatment within 48 h was significantly associated with admission to the ICU, mechanical ventilation and death (*P* < 0.05). The median day of virus detection turning negative was 3 days (range 1–11); however, these data were available only for 41 patients, because virus positivity was not rechecked in some patients with mild illness, and in some patients with severe illness, virus positivity was persistent even to death.

### Risk factors for admission to the ICU

Of the 111 patients, 14.4% (16/111) were admitted to the ICU. To explore the factors associated with ICU admission, we analysed all variables in Tables 1, 2, and 3, including the presence of an organ transplant (OR 4.00), diabetes (OR 4.94), the use of corticosteroids in the preceding 3 months (OR 3.32), neuraminidase inhibitor use before admission (OR 4.94), dyspnoea (OR 11.08), coinfection with other pathogens (OR 8.57), complications (OR 7.85), and no antiviral treatment within 48 h (OR 5.94). In the multivariate logistic regression model, dyspnoea (OR 32.33, 95% CI 2.07–504.56, *P* = 0.013), coinfection with other pathogens (OR 60.65, 95% CI 3.09–1188.84, *P* = 0.007) and no antiviral treatment within 48 h (OR 16.28, 95% CI 1.11–238.37, *P* = 0.042) were independently associated with increased risks of admission to the ICU (Table 6).

**Table 6:** Risks for admission to the ICU in immunosuppressed patients with influenza infections in logistics analyses

Variables	Odds Ratio	(95% CI)		*P*
**Univariate analysis**				
Organ transplantation	4.00	1.33	12.03	0.014
Diabetes	4.94	1.37	17.80	0.015
Use of corticosteroids in the preceding 3 months	3.32	1.12	9.87	0.031
Neuraminidase inhibitor use before admission	4.94	1.22	20.08	0.025
Dyspnoea	11.08	2.80	43.93	0.001
Coinfection with other pathogens	8.57	2.69	27.32	0.000
Complications	7.85	2.08	29.62	0.002
No antiviral treatment within 48 h	5.94	1.84	19.15	0.000
**Multivariate logistic regression analysis**				
Dyspnoea	32.33	2.07	504.56	0.013
Coinfection with other pathogens	60.65	3.09	1188.84	0.007
No antiviral treatment within 48 h	16.28	1.11	238.37	0.042

In the subgroup analysis of patients with malignancies receiving chemotherapy, coinfection with other pathogens (OR 45.71, 95% CI 4.21–496.51, *P* = 0.002) and complications other than pneumonia (OR 48.51, 95% CI 1.88–1248.98, *P* = 0.019) were independently associated with increased risks of admission to the ICU. In the subgroup analysis of patients who had undergone HSCT, dyspnoea was independently associated with an increased risk of admission to the ICU (OR 30.00, 95% CI 2.22–405.98, *P* = 0.011). None of the patients with immunosuppression for autoimmune diseases was admitted to the ICU.

### Risk factors for death

Among the 111 patients, 11 died. To explore the factors that were associated with death, we analysed all variables in Tables 1, 2, and 3, including the presence of an organ transplant (OR 7.00), the use of corticosteroids in the preceding 3 months (OR 3.80), neuraminidase inhibitor use before admission (OR 4.98), dyspnoea (OR 26.14), altered mental status (OR 13.83), complications (OR 17.14) and no antiviral treatment within 48 h (OR 8.64). In the multivariate logistic regression model, dyspnoea (OR 26.47, 95% CI 2.53–276.66, *P* = 0.006) and altered mental status (OR 68.15, 95% CI 2.88–1613.81, *P* = 0.009) were independently associated with increased risks of death (Table 7).

**Table 7:** Risks for death in immunosuppressed patients with influenza infections in logistics analyses

Variables	Odds Ratio	(95% CI)		*P*
**Univariate analysis**				
Organ transplantation	7.00	1.87	26.27	0.004
Use of corticosteroids in the preceding 3 months	3.80	1.07	13.56	0.040
Neuraminidase inhibitor use before admission	4.98	1.08	23.08	0.040
Dyspnoea	26.14	4.40	155.21	0.000
Altered mental status	13.83	2.28	83.86	0.004
Complications	17.14	2.11	139.64	0.008
No antiviral treatment within 48 h	8.64	2.26	33.03	0.011
**Multivariate logistic regression analysis**				
Dyspnoea	26.47	2.53	276.66	0.006
Altered mental status	68.15	2.88	1613.81	0.009

In the subgroup analysis of patients with malignancies receiving chemotherapy, altered mental status (OR 25.00, 95% CI 1.11–561.28, *P* = 0.043) was independently associated with an increased risk of death. In the subgroup analysis of patients who had undergone HSCT, dyspnoea was independently associated with an increased risk of admission to the ICU (OR 30.00, 95% CI 2.22–405.98, *P* = 0.011). None of the patients with immunosuppression for autoimmune diseases died.

## Discussion

In this study, nearly half of inpatients with influenza infection in PKUPH were immunosuppressed. Previous studies have mostly focused on influenza infection in patients who have undergone HSCT or SOT; however, more attention has been given to patients with immunosuppression due to other causes in recent years [4, 14, 15]. The most common cause of immunosuppression in this study was malignancies being treated with chemotherapy, which accounted for 64.0% of patients, including three patients with two different types of tumours. HSCT was the most common transplantation type. The median time of illness onset after HSCT was 9 months. Two-thirds of patients who had undergone HSCT experienced rejection in the preceding 3 months. Kumar et al. [11] reported that the median time of influenza infection in solid-organ transplantation patients after transplantation was 3.6 years, and the median time of influenza infection after HSCT was significantly shorter than that of solid-organ transplantation (Table 2). Nichols et al. [9] reported that 1.3% (62/4797) of HSCT patients were diagnosed with influenza virus infection within 120 days after transplantation, suggesting that influenza occurred early after HSCT and that these patients should pay more attention to the possibility of this illness.

The common presenting symptoms of influenza infection in immunosuppressed patients varied in different studies; however, most patients exhibited fever and cough [5, 16]. It is worth noting that fever and other classic symptoms, such as cough, sore throat, rhinorrhoea, headache and muscle soreness, were absent in immunosuppressed transplant recipients, whereas dyspnoea, gastrointestinal symptoms and altered mental status [5, 17], especially in HSCT patients, were reported in our study. Regarding complications, pneumonia was the most common complication (42.9%), consistent with previous literature (from 32 to 56%) [11, 14, 16]; however, other relatively rare complications, such as acute respiratory distress syndrome, shock, acute kidney injury, and viral encephalitis, should also be taken seriously. Coinfections with bacterial, fungal, and other viral pathogens were also found in the patients [3, 14, 16]. Coinfection with other pathogens, especially *Aspergillus spp.*, significantly prolonged the number of hospitalization days and increased the risk of admission to the ICU. The number of hospitalization days for two patients with *Aspergillus spp.* coinfection were 90 and 51 days, respectively (the latter patient died). Previous studies demonstrated that coinfections with influenza and *Aspergillus* were associated with a disruption of the bronchial mucosal barrier and phagocytosis, T cell dysfunction and apoptosis, neuraminidase inhibitor use and so on [18]. Complications and coinfections were also more common in HSCT patients than patients with immunosuppression due to other causes. Laboratory test results of influenza infection in immunosuppressed patients were characterized by lymphopenia. The median erythrocyte sedimentation rate and C-reactive protein were 31.5 and 27.7, respectively, which were slightly higher than normal. These results were partially consistent with those of previous studies, and lymphopenia was a risk factor for severe illness [19–21]. Thus, early identification of influenza infection in immunosuppressed patients is highly recommended. Fever and other classic symptoms may be absent in immunosuppressed transplant recipients, especially in HSCT patients. Conducting influenza virus detection at the first presentation seems to be a good choice for early diagnosis.

Many studies have suggested that clinicians start antiviral treatment as soon as possible for immunocompromised patients [15, 22, 23]. However, immunosuppressed patients were less likely to receive early antiviral treatment [24], which may be related to a delayed diagnosis due to the atypical clinical presentation of influenza immunosuppressed patients, especially HSCT patients. The symptom onset in the HSCT group was 60 h, which was significantly longer than that patients with immunosuppression due to other causes. Immunocompromised patients were susceptible to antiviral drug resistance due to prolonged viral shedding [8, 16, 25], which also contributed to more influenza-related deaths and nosocomial transmission [9, 26, 27]. Previous studies recommended higher doses and/or prolonged courses of antiviral therapy [7]. Combined and double doses of neuraminidase inhibitors did not significantly reduce the risk for admission to the ICU or death [28–30]. Patients did not benefit from the combination of oseltamivir and peramivir, possibly because peramivir binds to sialic acid residues in a manner similar to oseltamivir [31]. Therefore, a longer duration of antiviral treatment may be more beneficial for immunocompromised patients [13].

In our study, 14.4% of patients were admitted to the ICU, and the mortality rate was 9.9%, similar to that reported in other studies with larger samples [11, 14, 15]. The presence of an organ transplant, dyspnoea, the use of corticosteroids in the preceding 3 months, neuraminidase inhibitor use before admission, complications and no antiviral treatment within 48 h were associated with admission to the ICU and death in univariate analysis. In multivariate logistics analysis, risk factors for admission to the ICU were dyspnoea, coinfection with other pathogens and no antiviral treatment within 48 h, and the presence of dyspnoea and altered mental status were independently associated with increased risks of death. Some of the risk factors have been reported in previous studies [10, 11, 14, 32, 33], and some were first reported here. All analyses emphasized the identification of patients at risk of a severe course. Corticosteroid therapy may decrease inflammation at the expense of a longer duration of viral shedding [33–36].

Influenza-associated mortality in China was higher than that in other countries with higher vaccination coverage [37]. In the immunosuppressed population in our study, the vaccination rate was lower than 3%. The recommendations for influenza vaccines depend on the cause of immunosuppression [3]. Evidence has shown that an immunosuppressed patient could benefit from vaccination [34, 38–40]. The latest recommendation was to start inactivated influenza vaccines (IIVs) at 6 months after HSCT, and IIVs can be administered 3 months after transplantation in the setting of a community outbreak [41]. However, a systematic review showed that seasonal inactivated influenza vaccines remained suboptimal in patients who had undergone SOT [42]. There are no consensus guidelines on influenza vaccination for patients with malignancies, but the timing of vaccination was recommended to be more than two weeks before receiving chemotherapy or between chemotherapy cycles to enhance humoral responses [3].

In summary, our study provided important clues to understand the clinical characteristics of influenza infection in immunosuppressed patients, and influenza virus vaccines are highly recommended for this population. However, the study period was limited to five influenza seasons of one hospital, and some clinical data records were incomplete. For example, data for the median day of virus detection turning negative was available only for 41 patients because virus positivity was not rechecked in some patients with mild illness, and in some patients with severe illness, virus positivity was persistent even to death. Therefore, further multicentre randomized controlled studies with larger sample sizes will be needed to confirm and extend our findings.

## Conclusions

Our study analysed the clinical characteristics, risk factors and effects of antiviral therapy of influenza in immunosuppressed inpatients in China. Malignancies being treated with chemotherapy was the most common cause of immunosuppression, and more attention should be given to these patients. Fever and other classic symptoms may be absent in immunosuppressed recipients, especially in HSCT patients. Influenza virus detection in a timely manner allows early diagnosis. Patients did not benefit from combined and double doses of neuraminidase inhibitors. Patients with dyspnoea, altered mental status and coinfection with other pathogens were of note because these patients had a high risk of developing severe symptoms. The seasonal influenza vaccination rate in China is still low, and inactivated influenza vaccines should be highly recommended in immunosuppressed patients.

## Data Availability

The datasets used and/or analysed in the current study are available from the corresponding author upon reasonable request.

## Abbreviations

HSCT: Haemopoietic stem cell transplantation
μl: Microliter
SOT: Solid-organ transplantation
PKUPH: Peking University People’s Hospital
RT–PCR: Reverse transcriptase–polymerase chain reaction
ICU: Intensive care unit
WBC: White blood cell
BAL: Bronchoalveolar lavage fluid
G: 1,3-Beta-D-glucan
GM: Galactomannan
MRSA: Methicillin-resistant *Staphylococcus aureus*
IIVs: Inactivated influenza vaccines
